# Supplementary material for: Optimisation of AAV-NDI1 Significantly Enhances Its Therapeutic Value for Correcting Retinal Mitochondrial Dysfunction
Source: Pharmaceutics. 2023 Jan 18;15(2):322. doi: 10.3390/pharmaceutics15020322 (PMC9960502; doi:10.3390/pharmaceutics15020322)
Supplement: Supplementary file 1 [file pharmaceutics-15-00322-s001.zip › pharmaceutics-2059183-supplementary.pdf]

Suppl. Figures/Tables:

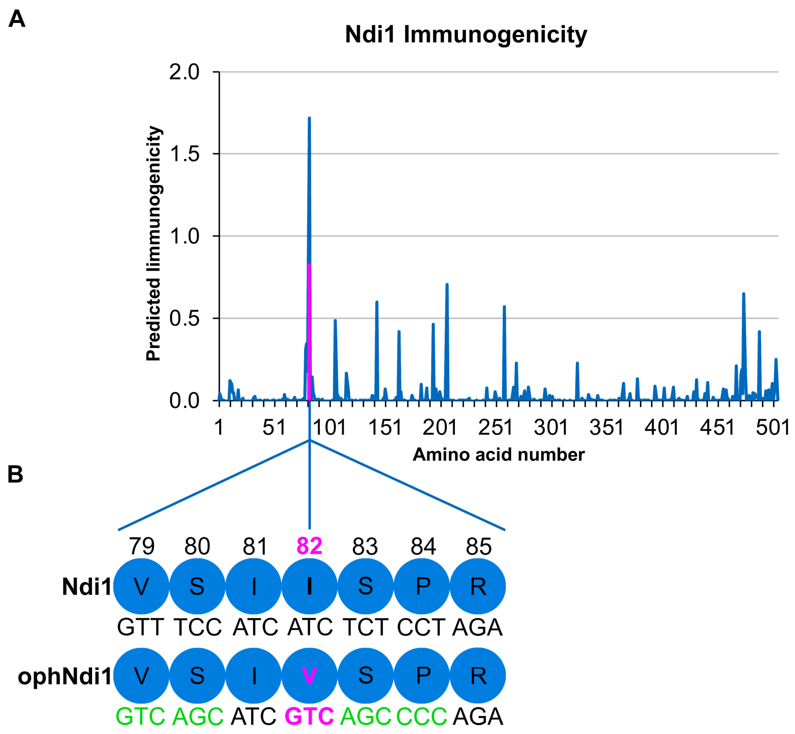

Figure S1. Optimisation of NDI1 (ophNdi1): (A). Graph of predicted immunogenicity of all 9-mer peptides in the protein using an IEDB proteasomal cleavage/TAP transport/MHC class I combined predictor, indexed by the position they appear in the peptide. Higher peaks indicate a greater likelihood of that 9-mer being presented by MHC-1 on the cell surface. (B). Example of codon optimisation. Blue circles represent amino acids, with their single letter code. Above is the amino acid position and below the codon triplet. Altered triplets that encode synonymous codons in ophNdi1 are highlighted in green. The amino acid substitution in ophNdi1, I82V, highlighted in pink and the pink line shows the reduced predicted immunogenicity of the valine substitution at position 82.

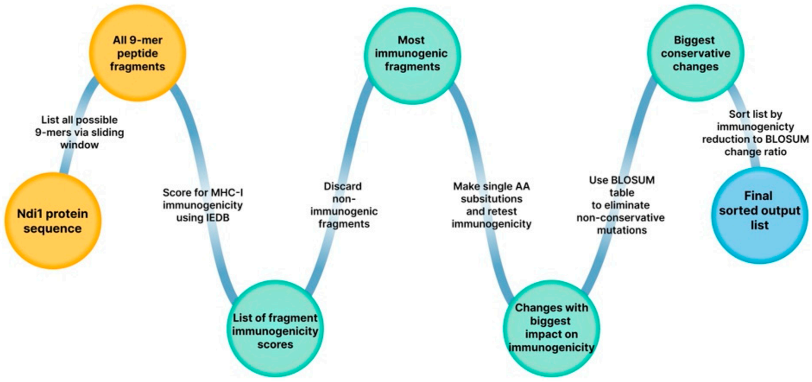

Figure S2. Optimisation of NDI1 (ophNdi1). Flowchart detailing the major steps in the immune-reduction pipeline. The full sequence of ophNdi1 can be found in patent no. 10220102.

Table S1. Optimal amino acid changes to reduce immunogenicity. Immunogenicity algorithm output listing potential amino acid changes in order of their predicted immunogenicity reduction (immune change, ΔS), scaled by the predicted disruption to the protein (BLOSUM change, ΔB); ΔS/ ΔB. The immune score is the total immunogenicity for all 9 of the 9-mers that contain a given amino acid. Initial: the amino acid at the position in the native protein, Position: position of the amino acid in the protein, New: replacement amino acid suggested by the program, Immunoscore: immunoscore for this locus in the native protein, Immuno change: change in immunoscore between the native and the modified locus, BLOSUM change: the change in BLOSUM score between the native and the modified position (lower numbers are more conservative).

Initial[1]	Position	New[1]	Immuno score	Immuno change (ΔS)	BLOSUM change (ΔB)	(ΔS) / (ΔB)
I	82	V	2.569	1.003	2	0.501
F	90	Y	1.926	1.497	3	0.499
L	89	I	2.104	1.254	3	0.418
V	266	I	0.667	0.363	1	0.363

[1] amino acid
